# Perception of patient safety culture among nursing students: A cross‐sectional study

**DOI:** 10.1002/nop2.1995

**Published:** 2023-10-19

**Authors:** Carmen Amaia Ramírez‐Torres, Félix Rivera‐Sanz, Ana Cobos‐Rincon, Teresa Sufrate‐Sorzano, Raúl Juárez‐Vela, Vicente Gea‐Caballero, Clara Isabel Tejada Garrido, Maria Estela Colado Tello, Angela Durante, Iván Santolalla‐Arnedo

**Affiliations:** ^1^ Department of Nursing University of La Rioja Logroño Spain; ^2^ Group of Research in Sustainability of the Health System Biomedical Research Center of La Rioja ‐ GISOSS (CIBIR) Logroño Spain; ^3^ Data Science, Big Data and Artificial Intelligence Unit. Healthcare Innovation; Group of Research in Sustainability of the Health System Biomedical Research Center of La Rioja–GISOSS (CIBIR) Logroño Spain; ^4^ Group of Research GRUPAC University of La Rioja Logroño Spain; ^5^ Group of Research in Sustainability of the Health System Biomedical Research Center of La Rioja (CIBIR) Logroño Spain; ^6^ I.P. Group of Research GRUPAC University of La Rioja Logroño Spain; ^7^ Faculty of Health Sciences Valencian International University Valencia Spain; ^8^ Research Group PBM Patient Blood Management, Health Research Institute IdiPAZ Hospital La Paz Madrid Spain; ^9^ Government of La Rioja Rioja Health Service, La Rioja Logroño Spain; ^10^ Group of Research INCUIDOS Biomedical Research Center of La Rioja (CIBIR) Logroño Spain; ^11^ I.P. Group of Research in Sustainability of the Health System Biomedical Research Center of La Rioja (CIBIR) Logroño Spain

**Keywords:** nursing, patient safety, patient safety environment, risk management, students

## Abstract

**Aim:**

To analyse the perception of patient safety culture among nursing students and to compare patient safety outcomes between the different year nursing groups.

**Design:**

A cross‐sectional descriptive study was conducted with nursing students (*n* = 266) between first and fourth years from one university in Spain.

**Methods:**

The project was conducted during the 2020/21 academic year. The data were collected using a translated and adapted version of the “Hospital Survey on Patient Safety” developed by the Agency of Healthcare Quality (AHQR).

**Results:**

Significant differences were found between the year of study of the nursing degree and whether or not specific training in patient safety culture had been received. The nursing students who had received specific training gave scores lower than anyone else in all questionnaire items, but only the indicators of “good practice” (*p* = 0.00) and “frequency of reported events” (*p* = 0.0012) showed significant differences. In some cases, fourth‐year students had lower significant mean scores in their “perception of patient safety within unit/sector,” “indicators of good practice” and “total score.”

**Public Contribution:**

Adverse events related to clinical practice continue to be a global problem. Improvements in patient safety require an increase in the patient safety culture of professionals and the promotion of development facilitators. Clinical practice and specific theoretical training foster greater awareness and demand related to patient safety, which is of interest when it comes to the development of new programmes that combine both methodologies and improve their effectiveness. Patient safety will continue to be a focus for all healthcare systems. The patient safety culture of future healthcare professionals should be developed at the university level in order to avoid unnecessary adverse events.

## 1 INTRODUCTION

Patient safety is a fundamental principle for healthcare systems (Ranotsi et al., 2022). In all healthcare processes there is a degree of risk inherent in the different activities involved (Fray et al., 2021; World Health Organization (WHO), 2019). The World Health Organization (2019) notes that adverse events are related to problems with clinical practice, its products, procedures and system. In total, 134 million adverse events occur each year in hospitals around the world. These events are the cause of a higher annual mortality rate (National Academies of Sciences, Engineering, & Medicine et al., 2018) and also cause a significant economic burden (Slawomirski et al., 2017).

Improving patient safety requires a complex system‐wide effort that encompasses a wide range of actions directed towards performance improvement; the management of safety and environmental risks, including infection control; the safe use of medicines; the safety of equipment; clinical practice and the environment in which care is delivered (Johnson et al., 2019). In addition, healthcare professionals themselves are bound by their code of ethics and by legislation to provide any technical and professional healthcare that suits the health needs of the people they serve (Alswat et al., 2017). An example is the Spanish Law 44/2003, of the 21 November, on Management of the Health Professionals which states that healthcare staff must develop their knowledge in line with established minimum levels of quality and safety. In this sense, it is essential that the principle of patient safety is incorporated into both academic and clinical quality improvement strategies (World Health Organization (WHO), 2011). Safety culture must be a central element within the organisational culture of healthcare facilities; embedding safety culture requires an understanding of the organisation's collective behaviour. The perceived experience of patient safety influences and changes attitudes towards care (Granel et al., 2020). For trainees, interactions with organisational processes and practices contribute to identifying and internalising the safety culture found in the different items of the organisation and transmitted to them through a process of enculturation (Jha et al., 2010) During the training process, both attitudes and behaviours are important elements acquired from the patient safety culture in an organisation and are modulated by the theoretical content acquired by the trainees. Specific patient safety training is essential when it comes to the competencies of the healthcare professionals. However, it needs to be integrated at university level and how to evaluate and improve this competence needs to be considered (Association of American Medical Colleges (AAMC), 2019).

### 1.1 University curriculum

In this School of Nursing, the theoretical content related to patient safety are distributed transversally among the different degree subjects. However, in a third year course, specific content related to patient safety is taught on its own in the “Quality of Care” module, which is an optional module taught over 4 months. The acquisition of a culture of patient safety is not usually included in the clinical practice teaching guides, in many cases only included guides in relation to the associated risks and/or effects derived from the administration and consumption of medicines. The extra‐curricular placements have a duration of 1950 h, distributed in the modular periods during second, third and fourth year.

The training of nursing students in patient safety is essential as nursing professionals are a key, decisive and very large group of professionals in every healthcare organisation (Huh et al., 2021).

Taking into account the need to improve patient safety culture, the aim of this study (to analyse the perception of patient safety culture among nursing students and to compare patient safety outcomes between the different year nursing groups) may lead to the introduction of academic reforms that aligns university training with national and international strategies.

## 2 METHODS

### 2.1 Design

A cross‐sectional, descriptive‐comparative study.

### 2.2 Participants and design

A purposive sample of students studying a Bachelor's Degree in nursing at the University of REDACTED during the 2020/21 academic year.

The study was conducted at the University of REDACTED School of Health Sciences. The reference population is defined by first to fourth‐year nursing degree students at the university (*n* = 298). The sample of 169 students produced a confidence level of 95% and a margin of error of 5%.

### 2.3 Data collection

#### 2.3.1 Recruitment

With both the School of Nursing's director's consent and the nursing degree lecturers' collaboration, the students were verbally informed of the study and were invited to participate by completing the questionnaire at the end of the last day of theoretical classes for each level. Thus, the data are collected for all the four student levels to enable the comparison of the results. The recruitment period was from January 2021 to March 2021 depending on the year and the last class and the questionnaire was provided on paper and filled in the usual classroom. After completing the questionnaires, they were delivered to a member of the research team.

#### 2.3.2 Hospital survey on patient safety culture for nursing students (HSOPS‐NS)

HSOPS‐NS was used to collect the data. The “HSOPS‐NS” tool, used by nursing students, is an adaptation of the Survey on Patient Safety Culture (SOPS) questionnaire designed by the Agency for Healthcare Research and Quality (AHRQ) (Sorra et al., 2019). The survey consisted of 54 items, each item was scored on a 5 point Likert scale consist of the below points: 1 being “strongly disagree,” 2 being “disagree” (DG), 3 being “neither agree nor disagree,” 4 being “agree” and 5 being “strongly agree.” However, the “individual perception of the patient safety environment” item was answered on a separate Likert scale (with 1 being “lacking” and 10 being “excellent”). The 54 items were divided into five items: “frequency of reported events,” “general perceptions of patient safety,” “perception of patient safety within the unit/sector,” “individual perception of patient safety environment” and “good practice indicators.”

Other socio‐demographic variables were considered, such as gender and age, year of study and whether they had taken the patient safety optional course.

### 2.4 Validity of tools used

The development and validation of the “HSOPS‐NS” corresponds to a research group from the Faculty of Medicine and Nursing at the University of the Basque Country and the Faculty of Health Sciences at the University of Zaragoza, which authorised this study. The factor analysis of the tool confirms a five‐factor solution that explained between 52.45% and 54.75% of the variance; the model determines an adequate fit CFI = 0.99; RMSEA = 0.05; Cronbach's alpha for the different items offers data between 0.74 and 0.77 (Ortiz de Elguea et al., 2019).

### 2.5 Data analysis

The data were analysed using the Statistical Package for Social Sciences (SPSS).

Descriptive statistics was conducted on the data: age; gender; year of study; specific training; frequency of reported events; general perceptions of patient safety; perception of patient safety within the unit/sector; individual perception of the patient safety environment and good practice indicators; absolute frequencies; means; percentages and standard deviation. The results were expressed in percentages and with 95% confidence intervals. The significance level was set at 0.05. The “*T* Student” and analysis of variance test was used for comparisons.

### 2.6 Ethical considerations

The study was approved by the Ethics Committee (REDACTED). All the nursing students signed informed consent to participate, as well as confidentiality agreements with the assurance their information would be used solely for teaching purposes and would be kept confidential.

## 3 RESULTS

### 3.1 Descriptive data of the sample

A total of 266 nursing students studying a nursing degree (89.26%) answered the survey. Seventy‐two (27.06%) were first‐year students, 70 (26.31%) were second‐year students, 81 (30.07%) were third‐year students and 43 (16.16%) were fourth‐year students. Of all the nursing students in the study, 62 (27.10%) had carried out specific training in patient safety (Table 1).

**TABLE 1:** Main socio‐demographic characteristics.

Variables	*n*	%
Gender		
Women	226	84.96
Men	40	15.03
Year of study		
1°	72	27.06
2°	70	26.31
3°	81	30.07
4°	43	16.16
Clinical quality and safety course		
Yes	62	23.30
No	204	76.69

In the terms of gender distribution, 225 were females (84.96%) and 40 were males (15.03%). The mean age of the participants was 21.62 years (standard deviation (SD) 4.57).

The analysis was redacted in two different ways. Firstly, by observing the items corresponding to the response frequencies as indicated by Ortiz de Elguea et al. (2019), and, secondly, an analysis of the means divided by the year of study and whether or not the optional subject of patient safety had been taken.

### 3.2 Analysis of items

#### 3.2.1 Frequency of reported events

The mean for this item was 3.56 (SD 0.96). For this item, the majority of students responded 4‐ or 5‐point Likert scale with statements saying that errors are recorded (66.9%).

#### 3.2.2 Overall perceptions of patient safety

The mean for this item was 3.38 (SD 0.52). For this item, the majority of the respondents scored either 4 or 5 on the Likert scale that patient safety was never sacrificed due to workload and that patient safety problems do exist within the unit (42.8%).

Additionally, the students mostly responded positively, either 4 or 5 on the Likert scale with aspects related to the supervision and management of actions to promote patient safety or teamwork within the unit (64.3%).

However, the majority of the respondents scored either 1 or 2 on the Likert scale with aspects related to open communication, such as staff feeling free to question decisions or actions taken by superiors (41%).

#### 3.2.3 Perceptions of patient safety in the unit

The mean for this item was 3.57 (SD 0.40). Respondents answered 4‐ or 5‐point Likert scale that the hours staff work in the units that they have been in may affect patient safety. However, they responded positively to other aspects related to staffing, supervisory support, inter‐unit teamwork and patient transfers and handovers.

#### 3.2.4 Individual perception of the degree of patient safety

The mean on this dimension was 8.12 (SD 1.01). The majority of the respondents scored an eight (41.2%), a nine (28.9%) or a seven (17.7%).

#### 3.2.5 Indicators of good practice

The mean for this item was 3.67 (SD 0.65). The majority of the respondents scored either 4 or 5 on the Likert scale that verbal treatment information was properly communicated with clear guidelines (69.9%), that changes in medication and diagnosis were communicated effectively (61.7%), and that patients were able to express their need for further explanation of complications or risk when signing an informed consent form.

#### 3.2.6 Total average on patient safety climate.

The overall mean for all items was 4.46 (SD 0.50).

### 3.3 Analysis of groups

#### 3.3.1 Nursing students by year of study

The differences in means between years of study is significant in all items except the “individual perception of the overall score on patient safety” (Table 2). The decrease in means is significantly different between first and fourth years in “frequency of reported events” with means of 3.86 and 3.29 (*p* = 0.014), respectively, “indicator of good practice,” with means of 3.90 and 3.29, respectively (*p* = 0.000); and “total score” with means of 4.61 and 4.23 (*p* = 0.002), respectively.

**TABLE 2:** Questionnaire means by year of study

Questionnaire item	Academic year			
	1st (SD)	2nd (SD)	3rd (SD)	4th (SD)
	(*n* = 72)	(*n* = 70)	(*n* = 80)	(*n* = 43)
Frequency of reported events	3.86 (1.35)	3.52 (0.72)	3.47 (0.79)	3.29 (0.76)
*p*	0.014*	0.213	0.081	0.014*
Perception of patient safety	3.39 (0.51)	3.27 (0.44)	3.52 (0.52)	3.28 (0.60)
*p*	1.00	0.032*	0.032*	1.00
Perception of patient safety within the unit/sector	3.60 (0.38)	3.46 (0.36)	3.70 (0.40)	3.43 (0.45)
*p*	0.04	0.003	0.003	0.04
Individual perception of patient safety environment	8.23 (0.98)	7.90 (1.01)	8.24 (1.08)	8.05 (0.89)
*p*	0.420	0.305	0.003	1.00
Indicators of good practice	3.90 (0.56)	3.80 (0.53)	3.59 (0.65)	3.29 (0.75)
*p*	0.000*	0.085	0.013*	0.000*
Total score	4.61 (0.51)	4.44 (0.47)	4.49 (0.46)	4.23 (0.53)
*p*	0.002*	0.411	1.00	0.002*

Abbreviation: SD, standard deviation.

* Statistical differences.

#### 3.3.2 Nursing students by specific training

The differences in means between specific or non‐specific training had no significant differences in “overall perceptions of patient safety,” “perception of patient safety in the unit or area” or “individual perception of the overall grade on patient safety” (Table 3). The decrease in the mean is significantly different between non‐specific and specific training in “frequency of reported events” with means of 3.32 to 3.59 (*p* = 0.019), respectively, “indicator of good practice” with means of 3.79 and 3.38 (*p* = 0.000), respectively; and “total score” with means of 4.51 and 4.33 (*p* = 0.015), respectively. We can hypothesize that specific training increases the perception of risk in all its fields.

**TABLE 3:** Means of the questionnaire on whether or not the student had taken the “Quality and Clinical Safety” course.

Questionnaire item	No specific training (SD) (*n* = 167)	With specific training (SD) (*n* = 62)
Frequency of reported events	3.59 (0.70)	3.33 (0.87)
*p*	0.019*	0.019*
Perception of patient safety	3.40 (0.55)	3.30 (0.51)
*p*	0.822	0.822
Perception of patient safety within the unit/sector	3.60 (0.39)	3.50 (0.45)
*p*	0.264	0.264
Individual perception of the patient safety environment	8.20 (0.78)	8.00 (1.14)
*p*	0.452	0.452
Indicators of good practice	3.79 (0.63)	3.38 (0.65)
*p*	0.000*	0.000*
Total Score	4.51 (0.46)	4.33 (0.50)
*p*	0.015*	0.015*

Abbreviation: SD, standard deviation.

#### 3.3.3 Nursing students by gender

The differences in means between gender was not significantly different in “frequency of reported events,” “overall perceptions of patient safety,” “individual perception of the overall score on patient safety,” “indicator of good practice means” and “total score.” The decrease in the mean was significantly different between women and men in only “perception of patient safety in the unit or area,” with means of 3.59 (SD 0.41) and 3.42 (SD 0.31; *p* = 0.025).

## 4 DISCUSSION

In the present study, fourth‐year students expressed less perception of patient safety than first‐year students, as did students who received specific training in patient safety than students who no received specific training. This study shows that both practical experience and training make students more critical and more aware of the elements they must assess when acting in a safe manner.

Although in the comparison between course, there is a significant decrease in terms of the means found for “indicator of good practice,” “total average on patient safety climate” and “frequency of reported events.”

The result that the averages in the items decrease as the courses progress has been found in other studies. It is considered to be related to a greater perception and awareness of the existence of risk generated by healthcare and, contrary to what one might think, the decrease in the averages can be considered an increase in the culture of patient safety.

Another key aspect of this reduction in results is the fact that clinical practice and real‐life interaction with healthcare services increases experience and therefore accountability (Huang et al., 2020).

Similarly, specific training allows students to become more aware of the relevance of patient safety, it improves the safety culture and reduces the scores on the questionnaire as the false sense of safety gradually disappears (Jha et al., 2010). In this vein, different studies report having observed differences when specific patient safety training is provided to groups of professionals, leading to an increase in reported adverse events and a change in attitude towards in the improvement of patient safety culture (Kong et al., 2019; Wanderlei & Montagna, 2018).

One of the items with statistical significance in this study, were both as the academic year progresses and specific training, is the frequency of reported events. According to Agency for Healthcare Research and Quality (2020) and Kong et al. (2019) factors such as “blame” or “punishment” as a result of reporting adverse events related to clinical safety decrease the likelihood of students and professionals will reporting such events (Agency for Healthcare research and Quality, 2020; Kong et al., 2019). Training in clinical safety and risk analysis favours a safety culture where they do not look for someone to blame, but rather they attempt to reduce and eliminate any risks in order to promote patient safety (Lee et al., 2020; Wanderlei & Montagna, 2018). In this case, we found similar results to those of Ortiz de Elguea et al. (2019), since first‐year students and, in our case, students without specific training are less familiar with the protocols and good practice guides, and this lack of knowledge could be surrounding them heightens the false sense of security (Ortiz de Elguea et al., 2019).

New advances in teaching methodologies allow for a greater combination of practice and theory that can lead to very positive results in routine aspects, such as adherence to patient safety strategies and encouragement of teamwork (King et al., 2018; McCoy et al., 2020). It is important to develop programmes that are directly linked to patient safety that enhance patient safety culture by fostering facilitators in professionals and avoiding potential barriers down the line (Association of American Medical Colleges (AAMC), 2019; Evans et al., 2014; Healt Quality Ontario, 2017).

The limitations of this study are due to the cross‐sectional and local design, as the findings. For future investigations, a prospective study should be used in order to observe the evolution of perception in patient safety in the same nursing students and avoid biases or external inclusions. On the other hand, the strengths of this study are come down to the sample size and the validated and translated tool for nursing students which increases the results' internal validity.

## 5 CONCLUSION

Future nursing professionals are an important driving force for change towards quality and patient safety in the healthcare system and in society itself. Specific training in clinical quality and safety will be offered throughout academic training during the nursing degree, as well as clinical practices, because studies have shown that students' perceptions of patient safety and healthcare risk are externally valid and critically situated. The increase in the safety culture of nursing students will determine an improvement to the quality of care, as well as to the safety standards.

## AUTHOR CONTRIBUTIONS

Conceptualization, C.A.R.‐T. and I.S.‐A.; methodology, R.J.‐V.; software, F.R.‐S. and V.G.‐C.; validation, T.S.‐S. and A.C.‐R.; formal analysis, C.I.T.‐G. and M.E.C.‐T.; investigation, A.D.; resources, I.S.‐A. and C.A.R.‐T.; data curation, R.J.‐V.; writing—original draft preparation, C.A.R‐T. A.C.‐R. and I.S.‐A.; writing—review and editing, C.I.T.‐G., M.E.C.‐T. and A.D.; visualisation, F.R.‐S. and V.G.‐C.; supervision, C.A.R.‐T. and I.S.‐A.; project administration, T.S.‐S.; funding acquisition, R.J.‐V., V.G.‐C. All the authors have read and agreed to the published version of the manuscript.

## FUNDING INFORMATION

We declare that we receive support by the University of La Rioja.

## CONFLICT OF INTEREST STATEMENT

The authors report no declarations of interest. This research did not receive any grant from funding agencies in the public, commercial or not‐for‐profit sectors.

## Data Availability

The data that support the findings of this study are available from the corresponding author upon reasonable request
